# Validity and usability of a professional association’s web-based knowledge translation portal: American Physical Therapy Association’s PTNow.org

**DOI:** 10.1186/s12911-015-0178-y

**Published:** 2015-10-08

**Authors:** Judith E. Deutsch, Wendy Romney, Jan Reynolds, Tara Jo Manal

**Affiliations:** Rivers Lab, Department of Movement and Rehabilitation Sciences, Rutgers University-School of Health Related Professions, 65 Bergen St. SSB 723, Newark, NJ 07101 USA; American Physical Therapy Association, 1111 North Fairfax St., Alexandria, VA 22314 USA; University of Delaware 160 STAR- Health Sciences Complex, Newark, DE 19713 USA

**Keywords:** Evidence-based practice, Usability, PTNow.org

## Abstract

**Background:**

PTNow.org is an evidence-based, on-line portal created by a professional membership association to promote use of evidence in practice and to help decrease unwarranted variation in practice. The site contains synthesis documents designed to promote efficient clinical reasoning. These documents were written and peer-reviewed by teams of content experts and master clinicians. The purpose of this paper is to report on the content and construct validity as well as usability of the site.

**Methods:**

Physical therapist participants used clinical summaries (available in 3 formats--as a full summary with hyperlinks, “quick takes” with hyperlinks, and a portable two-page version) on the PTNow.org site to answer knowledge acquisition and clinical reasoning questions related to four patient scenarios. They also responded to questions about ease of use related to website navigation and about format and completeness of information using a 1–5 Likert scale. Responses were coded to reflect how participants used the site and then were summarized descriptively. Preferences for clinical summary format were analyzed using an analysis of variance (ANOVA) and a Dunnett T3 post hoc analysis.

**Results:**

Seventeen participants completed the study. Clinical relevance and completeness ratings by experienced clinicians, which were used as the measure of content validity, ranged from 3.1 to 4.6 on a 5 point scale. Construct validity based on the information on the PTNow.org site was supported for knowledge acquisition questions 66 % of the time and for clinical reasoning questions 40 % of the time. Usability ratings for the full clinical summary were 4.6 (1.2); for the quick takes, 3.5 (.98); and for the portable clinical summary, 4.0 (.45). Participants preferred the full clinical summary over the other two formats (F = 5.908, *P* = 0.007). One hundred percent of the participants stated that they would recommend the PTNow site to their colleagues.

**Conclusion:**

Prelimary evidence supported both content validity and construct validity of knowledge acquisition, and partially supported construct validity of clinical reasoning for the clinical summaries on the PTNow.org site. Usability was supported, with users preferring the full clinical summary over the other two formats. Iterative design is ongoing.

**Electronic supplementary material:**

The online version of this article (doi:10.1186/s12911-015-0178-y) contains supplementary material, which is available to authorized users.

## Background

Evidence-based practice (EBP) integrates patient values, clinician expertise, and best available evidence to provide the best care [1]. Physical therapists (PTs) have acknowledged the importance of EBP in the American Physical Therapy Association (APTA) vision statement [2]. Physical therapists have a positive attitude toward using evidence [3, 4] and believe that using EBP is necessary; however, it has been difficult to implement the principles to inform examination and intervention [3–6].

Physical therapists and other health care professionals frequently report lack of time as the largest barrier to using evidence in practice [3–5]. Decreased confidence in literature search and appraisal skills is also a reported challenge [4]. In a survey of a random sample of 488 APTA members, Jette et al. [3] found that most agreed that EBP was necessary, that literature was helpful in their practices, and that the quality of patient care was better when evidence was used. However, 84 % indicated they needed to increase the use of evidence in their daily practice [3].

Knowledge translation (KT) and Knowledge to Action (KTA) frameworks provide a structure for assessing gaps in evidence-to-practice and for creating resources to fill those gaps [7]. KT strategies have been synergistically enhanced with the development of technology [8]. Evidence databases, specialized search engines, and website portals are available to help therapists’ access evidence more easily. The use of online resources has been associated with positive behavior change in health care workers including nurses, physicians, physical therapists, and occupational therapists [9–13].

**Fig. 1:**
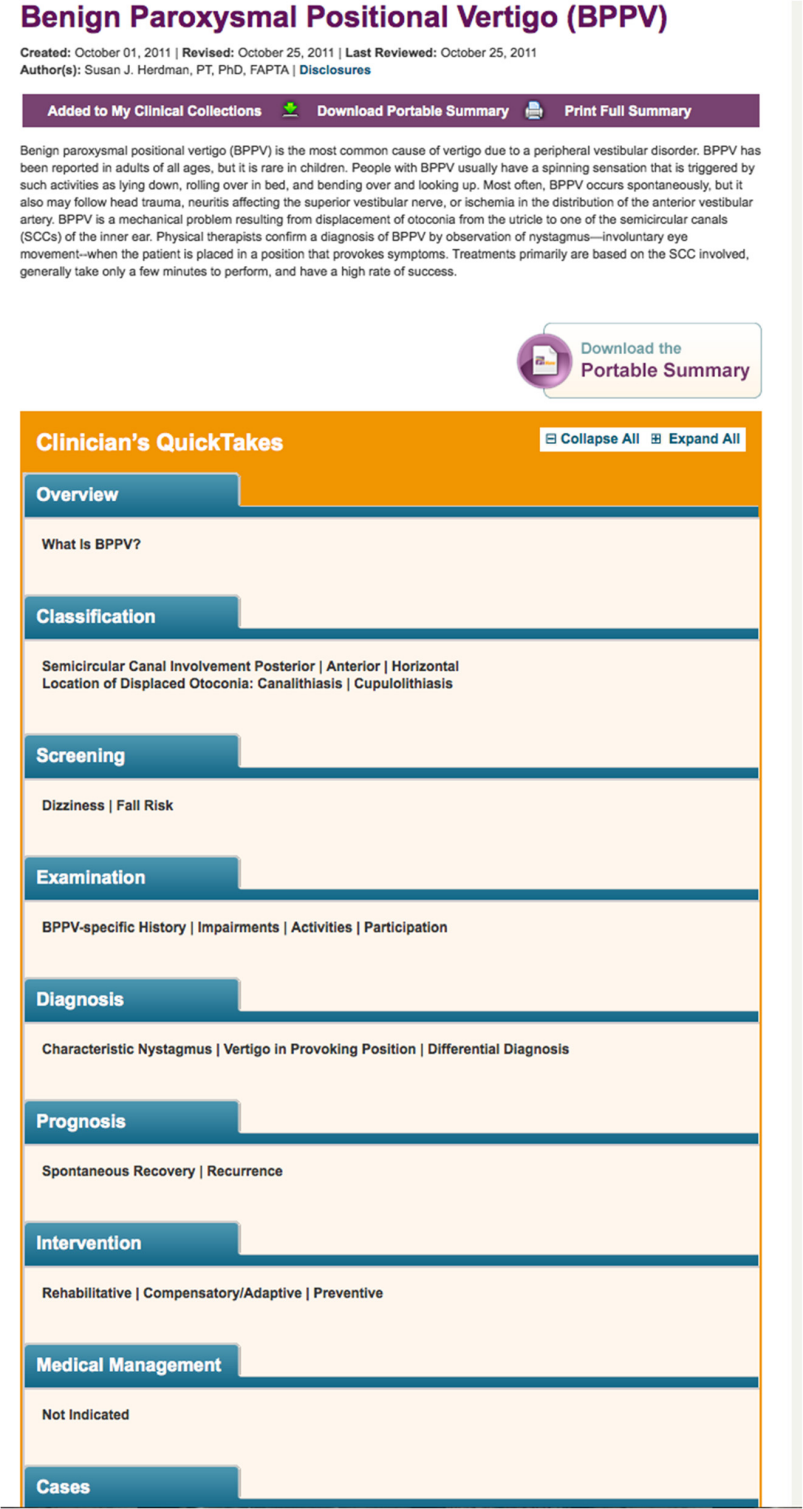
Example of Quick Takes

**Fig. 2:**
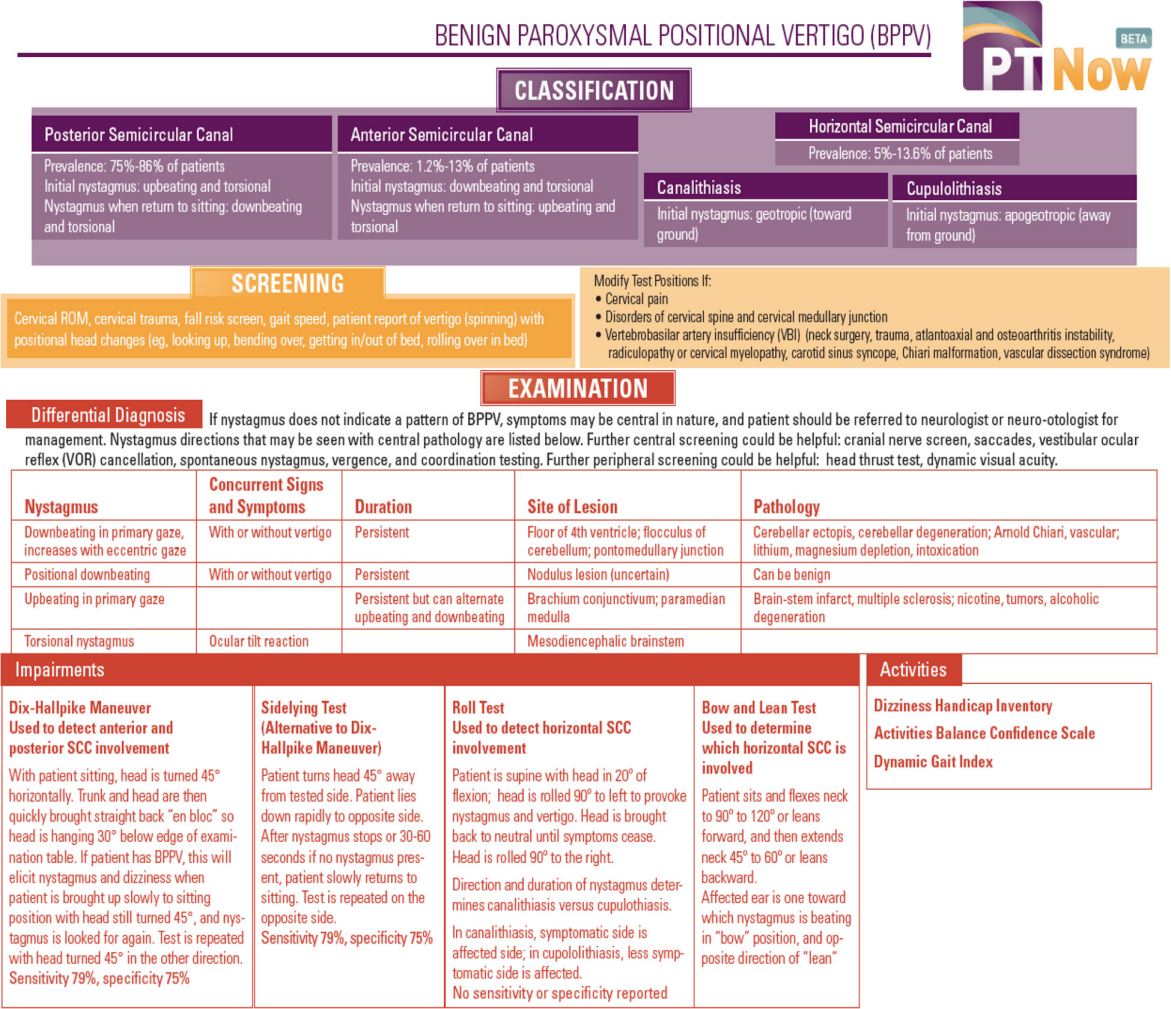
Example of portable summary (page 1)

The PTNow (PTNow.org) evidence-based portal was created by APTA to help reduce unwarranted variability in practice and promote the use of EBP. PTNow.org contains clinical summaries written and peer reviewed by clinician and scholarly experts. The clinical summaries aim to synthesize the literature in a clinically accessible manner organized by the patient/client management model from the *Guide to Physical Therapist Practice* [14]. There are three formats for the clinical summaries: full clinical summary, similar to a monograph, and "quick takes" (see Fig. 1) and a portable (pdf) clinical summary (see Fig 2). The clinical summary and "quick takes" are organized with tabs and hyperlinked to resources on and off the site; and the portable printable summary is a two-page precis.

Validity and usability are essential elements in guiding website design. Rehabilitation-focused evidence-based online resources such as Rehabilitation Measures Database [15] and StrokEngine have provided evidence for their content validity by asking users if the content was clinically relevant [13]. Additionally, both entities assessed site usability by having users comment on how easy it was to find and use the information on the site. However, construct validity for facilitating clinical reasoning was not established. Specifically, users’ knowledge or clinical reasoning skills were not tested in previous studies of on-line knowledge translation portals.

PTNow.org aims to be a knowledge synthesis resource that both provides content and guides clinical reasoning. A user-centered approach, querying focus groups of multiple constituents, informed the original design. Currently, iterative design and usability testing are refining the PTNow.org portal to ensure validity and make it useful and user friendly. Concurrently, site validity is being explored for content completeness (content validity) and interaction with the resources for gaining knowledge and making clinical decisions (construct validity). These steps are consistent with standard procedures whereby usability studies measure the effectiveness, efficiency, satisfaction, learnability, and accessibility of a new website [16]. The purpose of this study was to (1) provide evidence about content validity by users’ ratings of the resource’s clinical relevance and completeness (2) provide evidence about construct validity of participants’ knowledge acquisition and clinical reasoning by having them respond to patient scenarios using PTNow.org, and (3) report on participants’ perspectives on usability.

## Methods

### Participants

Practicing physical therapists were surveyed at two time points as part of the ongoing evaluation of the developing website. The first survey was conducted in 2012 and included questions about ease of use, knowledge acquisition, and clinical reasoning. Additional questions were added to the 2013 user study, including questions about internet use and clinical decision making in practice. Between the first survey in 2012 and the second survey in 2013, PTNow.org was enhanced with:

More links to outcome measures and psychometric information

Search ability using G-code categories for functional limitation reporting

New clinical summaries

Full-text clinical practice guidelines

Participants were recruited via email in the summers of 2012 and 2013. PTs who attended national PT conferences in 2011 and 2012 who provided PTNow their contact information were initially emailed. Additional participants were recruited by purposive sampling including Catherine Worthingham Fellows of the APTA (highest honor accorded to leaders in the field, who are typically consulted on association initiatives), members of the boards of directors of the Connecticut and Massachusetts state chapters of APTA, PT professional education faculty in Connecticut, and PTs certified by the American Board of Physical Therapy Specialties (ABPTS). PTs who indicated interest were sent a consent letter via email, which indicated that initiation of the survey signified consent. The letter contained links to the survey. On entering the online survey, participants were given a unique identification number. The study was approved by the Rutgers University Institutional Review Board.

### Patient scenarios

Once consented, participants were provided with links to four possible patient scenarios with the companion survey: benign paroxysmal positional vertigo (BPPV), chronic obstructive pulmonary disease (COPD), Parkinson disease (PD), and total knee arthroplasty (TKA). These scenarios were selected because they were the first clinical summaries posted on the PTNow.org site. The scenarios were written by one of the authors (JED) and reviewed for consistency by a second author (TJM). They contained comparable information and detail. Participants were asked to complete one or more patient scenarios and to use the clinical summaries on the PTNow.org site to answer corresponding questions. Each scenario was designed to take between 20 and 60 min to complete.

Patient scenarios consisted of a hypothetical case with a diagnosis of BPPV, PD, COPD, or TKA, and the participant was instructed to go to the PTNow.org site to answer questions related to the management of the case. These scenarios contained two parts. First, the patient was briefly described, and the study participant was asked to use PTNow.org to indicate how they would interview and examine the patient. After completing the knowledge acquisition questions, participants were then provided with information about examination results, and they were asked a set of clinical reasoning questions about direct patient care, patient/family education, and prognosis. To find out the format of the clinical summary that participants naturally selected (full clinical summary, quick takes and portable clinical summary), a forced choice was not imposed. An example of a patient scenario is below (see Additional file 1 for all of the patient scenarios and questions):

You are working as a home health care physical therapist, and you have just been assigned the case of a 65-year-old woman with PD (diagnosed 5 years ago and now in stage II of the Hoehn and Yahr Scale) who sustained a left hip fracture from a fall. She had her hip pinned and now is having some difficulties with bed mobility, transitional movements, ambulation in the home, and elevations. Her goals are for independence with bed mobility and with ambulation at home and in the community and for improved speed of movement. To formulate your examination strategy, you will use the information that you find in PTNow.org and the relevant resources in the clinical summary.

### Validity

Validity was evaluated based on responses to the clinical scenarios. Content validity captures whether the universe of information is represented and is specific to the content universe as defined by the researcher [17]. Content validity was operationally defined as the participant’s ratings about whether the information in the clinical summary was complete and useful in answering clinical questions. The responses were obtained exclusively from expert physical therapists who were clinical specialists (a certification based on years of experience and completion of an exam, awarded by the American Board of Physical Therapy Specialties [ABPTS]) or who had more than 5 years of clinical experience in practice.

The clinical reasoning construct was based on cognitive flexibility theory, wherein knowledge from different concepts and perspectives is re-constructed into an ensemble used to solve the current problem [18]. The “problem” in this study was the addressing of the questions associated with the clinical scenario. Construct validity was operationally defined as the response to knowledge and clinical reasoning questions based on using PTNow.org. Knowledge acquisition included questions related to patient interview and examination techniques as part of patient care. This construct captured general information that was required to answer the patient case questions; this information could be obtained directly from the clinical summary as well as the clinician’s prior knowledge. Knowledge acquisition responses were coded as *cut and paste*, *paraphrase*, or *prior knowledge*. Cut-and-paste responses were identical to the content in the clinical summary, paraphrase responses were similar to the content and organization of the clinical summary, and prior knowledge responses provided information that was not contained in the clinical summary or occurred when participants indicated that they did not use the clinical summary to answer questions in the scenario.

Clinical reasoning questions were on the topics of direct patient care, patient/family education, and prognosis based on examination findings provided in the scenario. Clinical reasoning questions required a level of synthesis and interpretation and were also evaluated for accuracy [19]. Coding for clinical reasoning questions was based on: 1) *Accuracy*, determined by the investigator based on a priori responses, 2) *Completeness*, which required a response and rationale, and 3) *Used the PTNow site material in the response*. Responses were coded as cut and paste, paraphrase, and prior knowledge, in the same manner as described above. Coding was checked for agreement between investigators WR and JD. In the event of a discrepancy the investigators discussed the issue and arrived at a consensus.

### Usability

Usability was assessed based on participants self-report. Participants rated their user experience on the site by responding to questions from the System Usability Scale (SUS) [20]. Participants were asked to rate on a five-point Likert scale (1 = completely disagree, 5 = completely agree) on the usability and content of each clinical summary format. Site usability was defined based on whether the information was (a) easy to find (navigation), (b) easy to interpret, (c) useful in answering questions, and (d) complete. In addition, open-ended questions were available for participants to discuss usability, completeness of the clinical summary, new information learned, and recommendations. Participants also responded to questions related to demographics, background, and their use of the internet.

### Data analysis

The demographic and survey responses data were analyzed descriptively, including frequencies, means, and standard deviations. Content validity based on the ratings of the clinical summary being complete and useful in answering the patient case was supported if the average ratings exceeded 4 out of 5. Construct validity was considered supported if it surpassed a threshold of two thirds of the responses (66 %) used the PTNow.org site, to answer the questions in the patient case. An ANOVA using SPSS Version 20.0 was conducted with a Dunnett T3 post-hoc analysis to investigate the differences in usability of the three formats of the clinical summary (full, quick takes and portable). A repeated measures ANOVA was not conducted because not all participants responded to each question and the data set, therefore, had to be treated as independent. An alpha level of .05 was set for all analysis.

## Results

One hundred and seventeen email invitations were sent to physical therapists in 2012 and 2013. Seventeen participants completed the usability study (9 in 2012, 8 in 2013). Six surveys were completed for the TKA scenario; 5 for BPPV; 2 for COPD; 2 for PD; and 2 for the comparison of PD and COPD.

Respondents had a mean age of 36.9 years (SD = 8.49 y), 81.25 % were female, and 43.75 % had received a master’s degree in physical therapy. They had practiced a mean of 12.93 years (SD = 9.18). All practice areas were represented, with the majority (38 %) practicing in an outpatient setting. Fifty six percent (56 %) were ABPTS-certified specialists, and 87.5 % were members of one or more APTA specialty sections (Table 1).

**Table 1:** Demographics of study participants

Demographics	2012 Survey (*n* = 9)	2013 Survey (*n* = 7)	Total *n* = 16^c^
Mean age (SD)	37.78 y	35.8 y	36.93 y (8.49)
Gender (%)			
Female	*n* = 7	*n* = 6	13 (81.25 %)
Male	*n* = 2	*n* = 2	
Mean years in practice (SD)	14.67	10.71	12.93 (9.18)
Highest degree (%)			
Master’s degree	*n* = 6	*n* = 1	*n* = 7 (43.75 %)
DPT^a^	*n* = 2	*n* = 4	*n* = 6 (37.5 %)
tDPT^b^	*n* = 0	*n* = 2	*n* = 2 (12.5 %)
PhD & tDPT	*n* = 1	*n* = 0	*n* = 1 (6.25 %)
Area of employment			
Hospital-based	*n* = 1	*n* = 1	*n* = 2
Outpatient	*n* = 2	*n* = 3	*n* = 6
Private Outpatient	*n* = 2	*n* = 0	*n* = 2
Skilled Nursing	*n* = 2	*n* = 2	*n* = 2
Academic Institution	*n* = 1	*n* = 1	*n* = 1
Other			
Specialization (%)			
Orthopedics	*n* = 3	*n* = 1	*n* = 4 (25 %)
Neurology	*n* = 0	*n* = 1	*n* = 1 (6 %)
Geriatrics	*n* = 3	*n* = 1	*n* = 4 (25 %)
Area of Expertise (%)			
Orthopedics	*n* = 5	*n* = 3	*n* = 8 (53 %)
Neurology	*n* = 0	*n* = 4	*n* = 4 (27 %)
Geriatrics	*n* = 3	*n* = 0	*n* = 3 (20 %)
Section member			
Education	*n* = 1	*n* = 1	n = 2
Geriatrics	*n* = 3	*n* = 0	*n* = 3
Neurology	*n* = 2	*n* = 3	*n* = 5
Orthopaedics	*n* = 2	*n* = 2	*n* = 5
Health Policy & Admin	*n* = 2	*n* = 0	*n* = 2
None	*n* = 1	*n* = 2	*n* = 3

^a^Doctor of Physical Therapy

^b^Transitional Doctor of Physical Therapy

^c^One participant did not complete the demographic information

### Internet use

All participants reported using the internet for professional activities, and 12 out of 15 reported using it for patient care. Eight participants reported using smart phones daily or weekly to assist with patient care. Of the eight participants who did not use smart phones, seven plan to use one in the future. Time spent on the internet, by activity, is reported in Table 2.

**Table 2:** Time spent on the internet by activity

	Mean hours (SD)	Range of hours per day
Internet activity	3.8 (3.5)	1.0-12.0
Internet use to assist with patient care	1.98 (3.2)	0-10.0
Internet use for other professional activity	1.5 (1.88)	0.1-8.0

#### Survey questions added in 2013

**Fig. 3:**
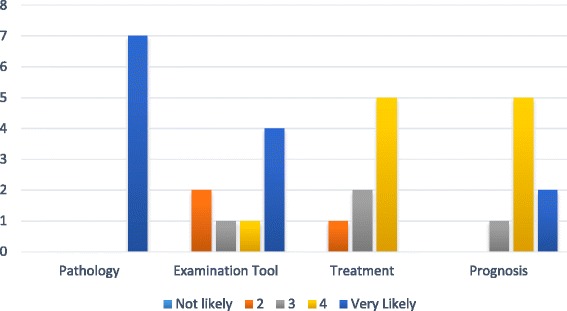
How participants report using the internet to guide patient management

In 2013, specific survey questions about how participants used the internet for patient care were added. The responses are summarized in Fig. 3. Participants reported using Google, *Physical Therapy,* PubMed, and Cochrane systematic reviews most frequently in clinical practice. Fifty percent reported that it was “easy” to “very easy” to find resources on the internet to guide patient care, but only 25 % thought it was easy to very easy to determine if the internet site was trustworthy. Participants believed their practice was between 10 % to 90 % evidence based. Access to trustworthy evidence was the most frequently reported barrier to EBP.

### Content validity: responses related to the completeness of the clinical summaries

Clinical experts (the participants with greater than five years of clinical practice experience or a specialty certification) rated content validity based on clinical relevance and site completeness. Experts rated the clinical relevance of the site by answering the question “Was the information on the site useful in answering clinical questions?” (on a scale of 1–5, with 5 being most complete) as follows, in means (standard deviations): full clinical summary, 4.5 (.82); quick takes, 3.0 (1.3); and portable summary, 3.67 (1.5). Clinical experts also rated the completeness of the clinical summary formats (on a scale of 1–5, with 5 being most complete) as follows: full clinical summary, 4.3 (0.8); quick takes, 3.1 (1.2); and portable clinical summary, 3.1 (1.3).

### Construct validity: responses to knowledge acquisition questions

A total of 32 knowledge acquisition questions were answered for the COPD, PD, and TKA clinical summaries. Participants used the clinical summary 66 % of the time to answer questions by cut-and-paste or paraphrase response strategy. Four participants used prior knowledge only to answer all knowledge acquisition questions, whereas eight participants used cut-and-paste or paraphrasing (from the content on the site) to answer knowledge-based questions. Only one participant used both prior knowledge and cut-and-paste to answer clinical questions (see Table 3).

**Table 3:** Responses for knowledge acquisition questions

	Cut and paste	Paraphrase	Prior knowledge	Totals
TKA	12	1	5	18
PD	2	2	4	8
COPD	2	2	2	6
TOTALS	16 (50 %)	5 (15.6 %)	11 (34.4 %)	32

*TKA* total knee replacement, *PD* Parkinson’s disease, *COPD* Chronic Obstructive Pulmonary Disease

### Construct validity: responses to clinical reasoning questions

A total of 42 clinical reasoning questions were answered for the COPD, PD, and TKA patient scenarios. Fourteen answers were coded as incomplete because participants provided no rationale. Twenty-seven of the 28 complete answers were correct. Of those, 40 % were based on the clinical summary (combining cut-and-paste and paraphrase answers) and 60 % used prior knowledge. To answer clinical questions, seven of the 12 participants used prior knowledge exclusively, and five of those seven had 15 years of clinical experience or more (see Table 4).

**Table 4:** Responses for clinical reasoning questions

	Cut and paste	Paraphrase	Prior knowledge	Totals
TKA	1	5	6	12
PD	0	2	6	8
COPD	2	1	5	8
TOTALS	3 (10.7 %)	8 (28.5 %)	17 (60.7 %)	28

*TKA* total knee replacement, *PD* Parkinson’s disease, *COPD* Chronic Obstructive Pulmonary Disease

### Site usability for 2012 and 2013

A total of 19 usability surveys (some participants completed more than one clinical summary) were completed. Usability of the TKA clinical summary was rated six times; BPPV, five times; COPD, four times; and PD, four times.

Participants used the full clinical summary 78.9 % of the time; quick takes, 57.9 % of the time; and the portable clinical summary, 26.8 % of the time. On average, participants “agreed” to “strongly agreed” that the full clinical summary was useful (4.6) and rated it highest in usability over the portable clinical summary and the quick takes formats. There was a significant difference in usability among formats (F = 5.908 and *P* = 0.007). Full summary was rated as more useful in comparison to the Quick Takes (Dunnett T3 = −1.06, *P* = 0.002) (see Table 5).

**Table 5:** Differences in Usability among the 3 Clinical Summary Formats^a^

	Quick takes (*n* = 11)	Portable summary (*n* = 7)	Full summary (*n* = 15)
	Mean (SD)	Mean (SD)	Mean (SD)
Overall mean rating	3.52 (0.98)	4.03 (0.65)	4.58 (1.2)
Useful	3.18 (1.17)	3.57 (1.40)	4.6 (0.74)
Easy to find	3.91 (0.70)	4.71 (0.49)	4.7 (0.45)
Easy to interpret	3.73 (0.65)	4.71 (0.49)	4.67 (0.49)
Complete	3.27 (1.19)	3.14 (1.43)	4.33 (0.82)

^a^1 = strongly disagree, 5 = strongly agree

### Responses to open-ended questions

**Fig. 4:**
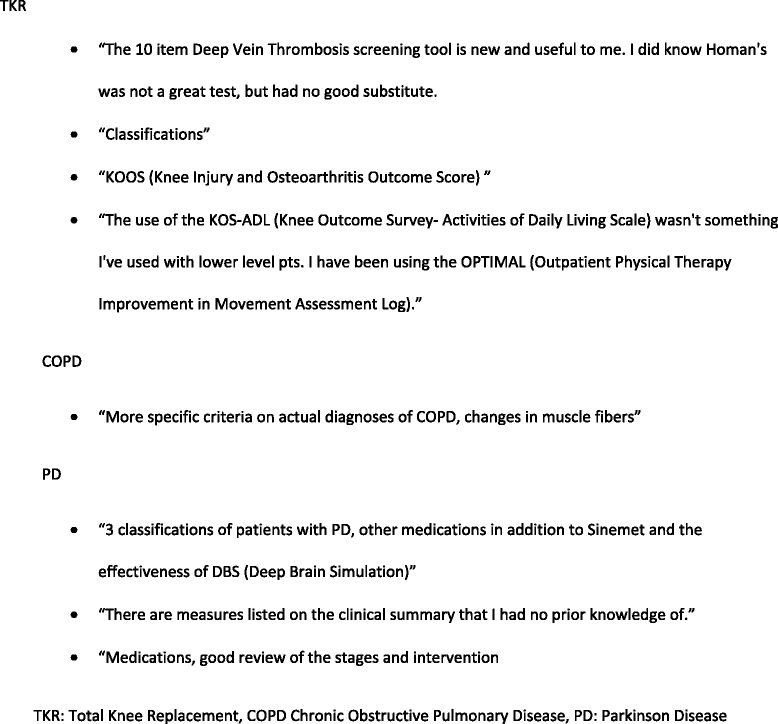
Comments about new or relevant information

**Fig. 5:**
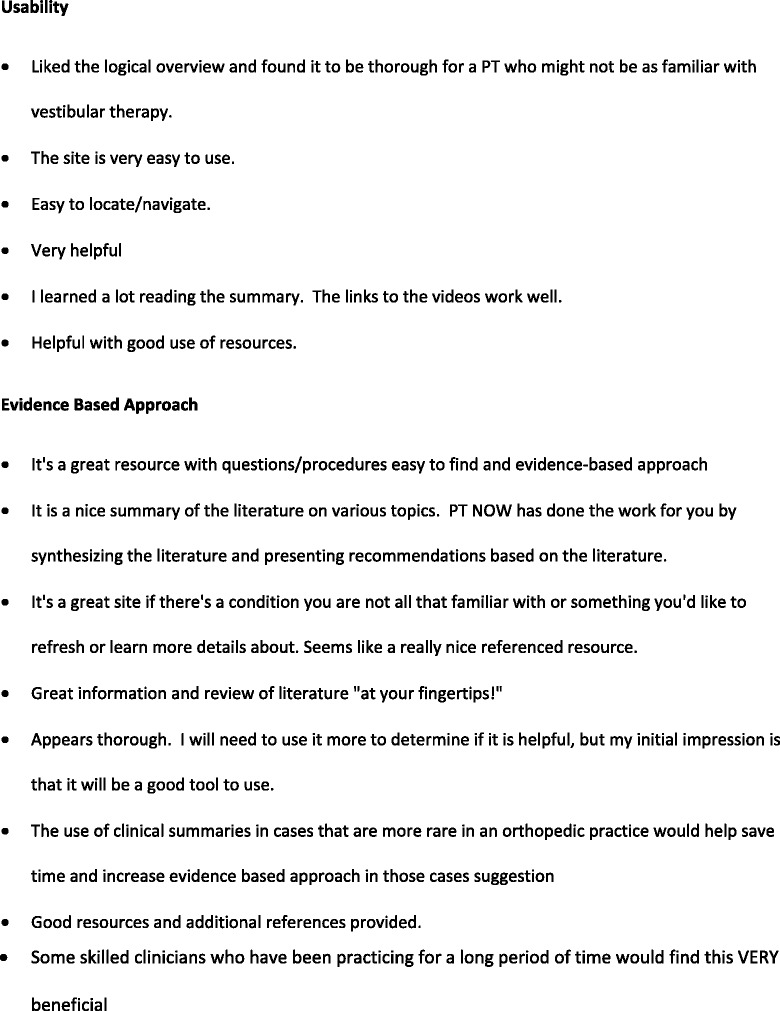
Comments about site usability and approach

Based on the responses to open-ended questions, 100 % of the participants would recommend the PTNow.org site to a colleague. More than 50 % of participants reported that, after reading the clinical summaries, they learned new or relevant information (Fig. 4). Information that was frequently reported as “new” was related to pathology, diagnosis, medications, and outcome measures. Participants reported that the site was easy to use, helpful, and evidence- based and that they enjoyed that the information was synthesized for the clinician. Fifty percent of the open-ended comments supported the approach of the site relative to evidence-based content and organization of information (Fig. 5). One user reported, “It’s a great site if there’s a condition you are not all that familiar with or something you’d like to refresh or learn more details about. Seems like a really nice referenced resource.” Participants also suggested improvements in site usability related to formatting and navigation. For example: “Move Full Clinical Summary and References to be seen towards the top of the page. I did not see it until I scroll all the way through the other sections. Putting it towards the right of the page instead of having ads would be beneficial.”

## Discussion

In this study we aimed to provide evidence about content and construct validity of PTNow.org and to determine participants’ perspectives on usability. Content validity was supported. Construct validity for knowledge acquisition was also supported. Construct validity for clinical reasoning related to patient scenarios using the PTNow site was partially supported. The site was rated as usable and useful with 100 % of participants reporting that they would recommend it to a colleague. Study participants preferred the full clinical summary, rating it 4.6/5 compared with a rating of 4.0/5 for the portable clinical summary and 3.5/5 for the quick takes.

### Construct validity: knowledge

Construct validity for knowledge was demonstrated based on the percent of responses that used cut-and-paste and paraphrase. The majority of participants used the clinical summaries to correctly answer knowledge acquisition questions. Further, in the open-ended responses, more than 50 % of participants reported learning something new or relevant. Evidence of knowledge acquisition is a common outcome for knowledge translation studies [21–23]. However, of greater interest to us was the use of the knowledge to make a clinical decision.

### Construct validity: clinical reasoning

Construct validity for clinical reasoning was only partially supported based on the questions that required clinicians to synthesize examination findings using the best available evidence and to make educated decisions about patient care [24]. Participants used prior knowledge (60 %) more frequently than paraphrasing (28 %) and cut-and-paste (12 %) to answer clinical reasoning responses. This may in part be explained by the level of experience of the clinicians (mean = 11 years) and the number (3/7) of clinical specialists. The fact that 28 % of the responses were paraphrased suggests that even experienced clinicians might have adapted their critical thinking based on interacting with PTNow.org. We speculated that with repeated exposure and familiarity with the resources, the paraphrasing strategy might increase. We speculate that testing people with less clinical experience may yield higher scores for construct validity.

### Usability

With the exception of quick takes, usability ratings were high for navigation and interpretation of information. Unexpectedly, participants preferred the full clinical summary and portable clinical summary over the quick takes. We had hypothesized that “quick takes”--which, unlike the portable clinical summary, is linked to additional resources such as tests and measures and patient education materials--might be found more useful than the portable summary. It is likely that, in the context of the study, participants chose to use a familiar monograph style. We speculate that in a clinical setting with a time limit, preferences for the 3 formats of the clinical summaries might differ from those indicated by this study. It is important to emphasize that we did not force a choice of format, but rather allowed the participant to select. This design choice provides insight into the participant’s preferences. The responses to the open-ended questions suggest that participants liked the format of the clinical summaries. They learned from the clinical summaries and found the organization logical. In the context of the current study, the usability of the clinical summaries was supported.

Our findings are comparable to previous work on knowledge translation resources designed for rehabilitation that were tested for content validity and usability. Usability testing of StrokEngine found users to be “very to extremely satisfied” with layout/organization, quality, and clinical relevance [13]. Interestingly, this group also reported that StrokEngine had significantly higher usability scores (mean = 43, SD = 4) (*P* < 0.005) than Cochrane Reviews Database (mean = 26, SD = 8), Royal College of Physicians (mean = 20, SD = 5), and general internet search (mean = 26, SD = 7) [13]. The Rehabilitation Measures Database usability testing found the site easy to use and that information on the site was relevant to participants [15]. Our work, however, presents novel information on construct validity that relates to clinical reasoning.

### Study design considerations

The number of participants studied is relatively small (*n* = 17); however, in usability studies, the number of participants may be small because of the task requirements and the iterative requirements for design [25]. We believed that a sample of 17 response sets was a reasonable representation based on the number of patient scenarios, the tasks required of the participants, and our plans for future user studies. The lack of a forced choice meant that, in some instances, a clinical summary format was not used and therefore not evaluated. Further study on the clinical summary format--in particular, the usefulness of quick takes--is indicated. In addition, we had to code clinical questions as incomplete when rationale was not provided. A “talk aloud” usability study would both (1) allow directing a participant to select all aspects of the site and (2) gain insights into participants’ choices of clinical summary format and insights into their clinical reasoning.

Although this type of user study is designed to create a scenario where clinicians visit and interact with a website based on clinical behavior, this is still not a true clinical situation. Usability studies are planned to determine the clinical usability in the natural setting. This will require that the site undergo responsive design, permitting the user at the point of care to access PTNow.org either on a tablet or a cell-phone.

## Conclusion

Based on the results of this study, clinicians of different specialties and from different work settings were able to use the clinical summaries to answer knowledge acquisition and clinical reasoning questions related to the scenarios, thus providing preliminary evidence on content and construct validity. The PTNow.org site was created with multiple formats to meet the different needs of physical therapist clinicians. The results of this usability summary validate and support the design and approach.

## Additional file

Additional file 1:
**Patient Scenarios and Questions.**

## Abbreviations

PT: Physical therapy, physical therapist
APTA: American Physical therapy Association
BPPV: Benign paroxysmal positional vertigo
COPD: Chronic obstructive pulmonary disease
PD: Parkinson disease
TKA: Total knee arthroplasty
EBP: Evidence-based practice
ABPTS: American Board of Physical Therapy Specialists
DPT: Doctorate of Physical Therapy
tDPT: Transitional Doctorate of Physical Therapy
