# Supplementary material for: Validity and usability of a professional association’s web-based knowledge translation portal: American Physical Therapy Association’s PTNow.org
Source: BMC Med Inform Decis Mak. 2015 Oct 8;15:79. doi: 10.1186/s12911-015-0178-y (PMC4599310; doi:10.1186/s12911-015-0178-y)
Supplement: Additional file 1: — Patient Scenarios and Questions. [file 12911_2015_178_MOESM1_ESM.pdf]

Additional Files

COPD User Scenario

You are a home health care physical therapist, and you have just been assigned the case of a 65-year-old man with COPD (diagnosed 15 years ago and now at the moderate stage) who sustained a right hip fracture from a fall. He had his hip pinned and now is having some difficulties with bed mobility, transitional movements, ambulation in the home, and elevations. His goals are to return to work in an office with walking requirements of about 1,000 meters per day. To formulate your examination strategy, you will use the information that you find on the PTNow site along with the relevant resources in the clinical summary.

Once you've logged in, click on the Clinical Summaries tab, then browse for COPD and click on it.

Once in the COPD clinical summary, you will see that it has different sections you can select—Clinician's Quick Takes, Full Clinical Summary, and Portable Summary. Please gather information for your first clinical examination.

Based on what you find on the site, please answer the following questions:

What interview questions will you ask?

What examinations will you perform?

What other relevant information did you find?

Pretend that you have now conducted the examination, with these results:

History of HTN and sleep apnea

- History of falls about 1 month, typically when he gets out of bed to go to the bathroom at night
- He is using a short-acting bronchodilator

• Hip pain: 5/10 with elevations, 3/10 with walking

• Weakness in both lower extremities

• Timed “Up and Go”: 25 seconds with a cane on the left

• Six-minute walk test: unable to complete

• Wife supportive and available

<u>What is the prognosis?</u>

<u>Patient and family education?</u>

<u>What Direct Care will you provide?</u>

<u>TKA User Scenario:</u>
You practice in an outpatient orthopedic setting. You are evaluating a patient who is 2 weeks post bicompartmental, posterior cruciate ligament-sparing TKA. Following surgery, she received home physical therapy for 1 week, focusing on ROM, patellar mobilization, knee extension exercises, and pain and effusion management. She has a history of diabetes and currently is a smoker (1 pack/day). She ambulates with a rolling walker and reports difficulty negotiating stairs and rising from a chair. To formulate your examination strategy, use the information that you find in PTNow and the relevant resources in the clinical summary to formulate your examination strategy.
Once you’ve logged in, click on the Clinical Summaries tab, then browse for TKA and click on it.
Once in the TKA clinical summary, you will see that it has different sections you can select—Clinician’s Quick Takes, Full Clinical Summary, and Portable Summary. Please gather information for your first clinical examination.

1. Based on what you find on the site, please answer the following questions:

What interview questions will you ask?

What examinations will you perform?

What postoperative conditions is this patient at risk for?

What other relevant information did you find?

Pretend that you have now conducted the examination, with these results:

- Pain rating within past 24 hours: best - 8/10, worst - 9/10
- ROM: lacking 10 degrees – 90 degrees
- Patellar mobility: hypomobile in all directions
- Quad set: fair with minimal superior glide of patella
- Quad strength: 70% strength deficit compared with uninvolved leg
- Inspection of incision: erythema surrounding incision, increased tissue temperature, drainage noted on bandage
- Timed "Up and Go": 20 seconds with rolling walker
- Knee Outcome Score-Activities of Daily Living Scale: 50%

Use the clinical summary and the information provided above to guide you as you plan your first intervention. Please explain in writing what you will do to address:

Patient and family education

Direct Care

Should you contact the physician?

Why/Why Not?
